# Remote ischemic conditioning for safety, feasibility and preliminary efficacy of patients with aneurysmal subarachnoid hemorrhage after aneurysm clipping, design, and protocol for an open-label, evaluator blinding randomized controlled trial

**DOI:** 10.3389/fneur.2025.1650773

**Published:** 2025-11-20

**Authors:** Liuyu Xu, Tonghu Jin, Hao Niu, Yunjian Yin, Kuang Yan, Hao Guan, Aihua Liu

**Affiliations:** 1 Beijing Neurosurgical Institute, Beijing Tiantan Hospital, Capital Medical University, Beijing, China; 2 Beijing Institute for Brain Disorders, Capital Medical University, Beijing, China; 3 People's Hospital of Ningxia Hui Autonomous Region, Ningxia Medical University, Yinchuan, Ningxia, China

**Keywords:** remote ischemic conditioning, aneurysmal subarachnoid hemorrhage, stroke, protocol, brain

## Abstract

**Background and rationale:**

Aneurysmal subarachnoid hemorrhage (aSAH) is a severe cerebrovascular event with a high mortality and disability rate. Compared to interventional surgery, microsurgical clipping is more invasive and has a higher incidence of postoperative complications, including cerebral vasospasm and ischemic cerebral infarction. In recent years, a large number of basic experiments have proved that remote ischemic conditioning (RIC) has multiple pathways of neuroprotective effects, and many large-scale randomized controlled clinical trials have confirmed that remote ischemic conditioning applied to patients with ischemic stroke has the effect of improving prognosis. However, current research has only focused on demonstrating the safety of remote ischemic conditioning for patients with aSAH, and lacks high-level evidence for its effectiveness.

**Method:**

We design an open-label, evaluator blinding randomized controlled trial. This study focuses on aneurysmal subarachnoid hemorrhage occurring within 24 h after microsurgical clipping. All participants will be randomly assigned to the intervention group and the control group at a 1:1 ratio (*n* = 20), and will receive standard management according to the guidelines. Participants assigned to the intervention group will receive RIC twice a day, once in the morning and once in the afternoon, for 7 consecutive days after the operation. Neurological prognosis will be evaluated at baseline, day 3, day 7, day 30, and day 90. The primary outcome measure is the proportion of patients with a mRS score of 0–2 at 90 ± 7 days after surgery and the incidence rate of RIC adverse events. The secondary measures include the incidence of aSAH complications, the mRS score of patients 30 ± 7 days after surgery, and the mRS score of patients 7 ± 1 day after surgery.

**Discussion:**

The study’s aim is to explore the safety, feasibility and preliminary efficacy of RIC in aSAH patients after clipping surgery. It serves as a methodological and feasibility foundation for the later multicenter, double-blind REPAIR trial (NCT06711302), which aims to confirm efficacy in a larger population.

**Clinical trial registration:**

https://clinicaltrials.gov/study/NCT06819657?term=NCT06819657&rank=1, NCT06819657.

## Introduction and rationale

Subarachnoid hemorrhage (SAH) is a severe neurological disorder, with an overall incidence of approximately 9 cases per 100,000 people per year (1, 2). About 85% of non-traumatic subarachnoid hemorrhages are caused by aneurysm rupture (3, 4). Aneurysmal subarachnoid hemorrhage may also be accompanied by neurological complications such as cerebral vasospasm, hydrocephalus, delayed cerebral ischemia, and re-bleeding of the aneurysm (5–7), with a relatively high mortality and disability rate (8). Currently, there are two main treatment methods for aneurysmal subarachnoid hemorrhage worldwide: craniotomy microsurgical clipping and endovascular interventional therapy. According to a meta-analysis, microsurgical clipping has more advantages in closing the aneurysm and reducing the risk of re-bleeding, but it may increase the risk of adverse outcomes and vasospasm (9). In recent decades, although many treatment strategies, such as electrolyte management, and intracranial pressure management, have been studied and widely applied in postoperative management, aSAH patients still have difficulty achieving a good prognosis (10). Therefore, there is an urgent need for new aSAH treatment strategies and methods in clinical practice.

Remote ischemic conditioning (RIC) is a non-invasive treatment for distant organs or tissues (such as the limbs) that involves brief, repetitive ischemia and reperfusion (11). It is a safe and well-tolerated treatment for cardiovascular and cerebrovascular diseases. Numerous large randomized controlled clinical trials have shown that RIC can improve the prognosis of ischemic stroke patients and prevent ischemic stroke in patients with intracranial atherosclerotic stenosis (12–14). Additionally, some basic experiments have demonstrated that RIC can regulate the differential gene expression and inflammatory response processes in mice with subarachnoid hemorrhage, and reduce the incidence of cerebral vascular spasm and improve cognitive dysfunction in experimental mice (15, 16). In patients with aneurysmal subarachnoid hemorrhage, RIC has been proven to be safe and feasible, with the potential to prevent delayed cerebral vasospasm and reduce the incidence of stroke and mortality (17–19). Although previous studies have explored the feasibility or early clinical impact of remote ischemic conditioning (RIC) in aneurysmal subarachnoid hemorrhage (aSAH), high-quality randomized controlled evidence confirming both safety and efficacy after surgical clipping remains limited (17, 20, 21). Therefore, we designed a double-center, open-label, evaluator-blinded pilot randomized controlled trial (NCT06819657) to apply remote ischemic conditioning to aSAH patients who have undergone microsurgical clipping, aiming to explore the safety, feasibility, and preliminary efficacy of unilateral lower limb remote ischemic conditioning in aSAH patients, providing good guidance and reference for the later multicenter, double-blind REPAIR trial (NCT06945239).

## Methods

### Study design

**Figure 1:**
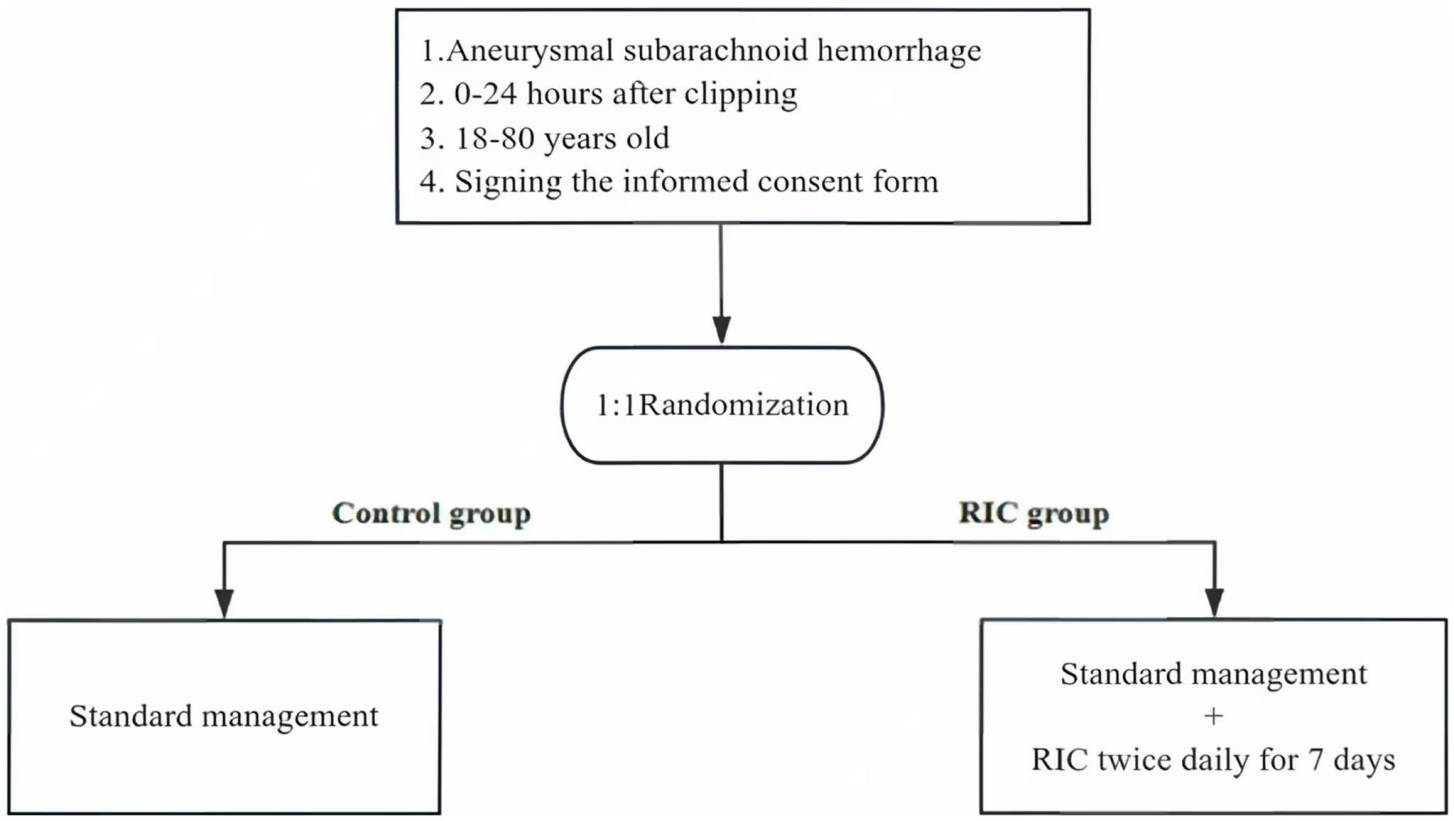
Trial design flowchart. RIC, remote ischemic conditioning.

We conduct an open-label, evaluator blinding, randomized controlled clinical trial in People’s Hospital of Ningxia Hui Autonomous Region and Beijing Tiantan Hospital on patients with aSAH who have undergone microsurgical clipping. Subjects who meet the inclusion criteria and do not meet the exclusion criteria will be randomly assigned to the RIC group and the control group within 24 h after microsurgical clipping, as shown in Figure 1. Both groups of subjects will undergo standardized and standardized management. The subjects in the RIC group will undergo remote ischemic preconditioning for 7 consecutive days after enrollment, once in the morning and once in the afternoon each day. The National Institutes of Health Stroke Scale (NIHSS) score will be blindly evaluated by trained researchers at the baseline. The Modified Rankin Scale (mRS) will be blindly evaluated by trained researchers on the 7th, 30th, and 90th days after enrollment.

All the participants will be enrolled after being fully informed of the content of this clinical study and signing the informed consent form. This study has been approved by the ethics committees of each research center and has been registered on Clinicaltrials.gov, with the registration number being NCT06819657.

### Inclusion and exclusion criteria

Participants will be recruited from the wards. The inclusion criteria are: 1. Imaging examination confirmed aneurysmal subarachnoid hemorrhage. 2. Responsible aneurysms received craniotomy clipping within 24 h. 3. 18 ≤ age ≤80 years old.4. Informed consent of the participant or legally authorized representative. The exclusion criteria are: 1. Patients with other types of cerebral hemorrhage. 2. Prior neurological impairment (mRS Score >1) or mental illness may confuse neurological or functional assessment. 3. Severe comorbidities with a life expectancy of less than 90 days.4. Refractory hypertension (systolic blood pressure 180 > mmHg or diastolic blood pressure 110 > mmHg). 5. RIC contraindications: severe soft tissue injury of lower limbs, peripheral arterial disease or coagulopathy. 6. Simultaneously participate in another research program to study a different experimental therapy. 7. Any condition that the investigator believes may increase the patient’s risk.

### Randomization

In this study, a computer-generated randomization sequence is used to allocate 40 participants to the RIC group and the control group (20 participants in each group) in a 1:1 ratio. Subsequently, the research assistant, who is not involved in the study, seals the group information in sequentially numbered opaque sealed envelopes according to the randomization sequence. All the random envelopes are uniformly stored in a confidential document cabinet before the start of the trial. After completing the participant enrollment screening and the collection of baseline data, the treating physician opens the random envelopes corresponding to the serial numbers and carries out the grouping operation according to the information contained therein.

### Intervention

**Figure 2:**
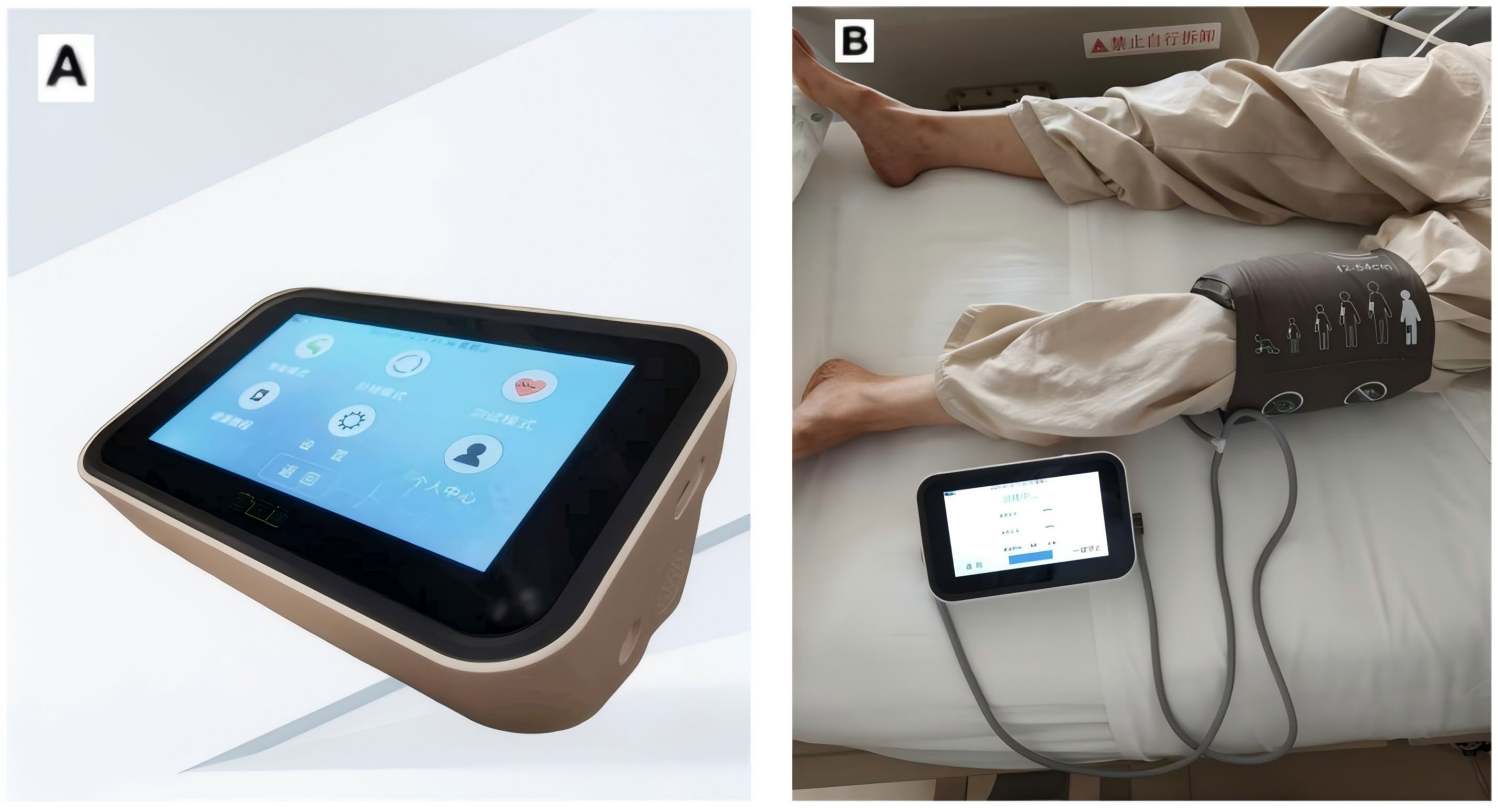
The equipment used by the research. **(A)** Patent number: ZL2014108343052; device model: IPC-906H; Beijing Renqiao Cardiocerebrovascular Disease Prevention and Treatment Research Jiangsu Co., Ltd. **(B)** Remote ischemic condition performed in human.

Both groups of participants will receive standardized and standardized treatment. In addition, the subjects assigned to the RIC group will undergo RIC procedures once in the morning and once in the afternoon each day after enrollment, for a total of 7 days. At the two research centers, the RIC procedures will be carried out by using the same electric automatic control device (placing the cuff on one lower limb as shown in Figure 2), with the cuff inflated to 200 mmHg for 5 min, followed by re-inflation for 5 min, for a total of 5 cycles per session. The RIC procedures will be conducted under the assistance of hospital nurses. During the operation of the RIC device, the screen does not display the pressure applied by the cuff. We selected the IPC-906H device because it’s approved by the National Medical Products Administration (NMPA) in China for cerebrovascular clinical research. To ensure consistency and reproducibility, we used the same model across two participating centers.

### Outcome

#### Safety outcomes assessment

The safety evaluation indicators are: (1) Mortality rate within 90 days. (2) Adverse events related to RIC (including limb skin injury, local pain, and deep vein thrombosis).

#### Primary outcome measure

The primary outcome measures were the proportion of patients achieving a good functional outcome (mRS score 0–2) at 90 ± 7 days after surgery, and the incidence rate of RIC-related adverse events. The primary endpoint was assessed by blinded neurologists who were not involved in treatment allocation, using standardized interviews.

#### Secondary outcome measures

1. The incidence of aSAH complications.
2. The mRS score of patients 7 ± 1 day after surgery.
3. The mRS score of patients 30 ± 7 days after surgery.

This study adopts a blinded assessment design to ensure the objectivity of the data. The data collection and processing flow is as follows: All clinical data at baseline and during the follow-up period are collected by the attending physician and his team. The subjects are assigned a unique code (001-040) according to the order of enrollment. The core outcome indicators are verified by two research coordinators who are not involved in the management of the subjects and remained blinded to the group information, using a double-blind back-to-back assessment model to review the original data. If there are differences during the assessment, they will first be resolved through reviewing the original records and standardized judgment guidelines; if there are still differences, they would be submitted to a third independent assessor for arbitration. Finally, the result data and grouping information will be collected by a researcher and subjected to statistical analysis.

### Data monitoring

An independent Data Safety Monitoring Board (DSMB) consisting of one neurosurgeon, one neurologist, and one statistician not involved in the study will oversee data integrity and participant safety. The DSMB will review recruitment rates, adverse events, protocol adherence, and data completeness every three months through both remote and on-site monitoring. All serious adverse events will be reported within 24 h to the DSMB and ethics committees.

### Sample size estimation

Previous studies have shown that a sample size of 10-20 cases per group in a randomized controlled clinical trial is sufficient to fully meet the requirements (22) for feasibility assessment (including core parameters such as recruitment efficiency and intervention compliance). This range is in line with the efficacy calculation standards for pilot studies, and can effectively balance the requirements of scientific research ethics and resource optimization allocation. Assuming a 60% reduction in poor outcomes (mRS>2) in the RIC group compared with controls at 90 ± 7 days after surgery, the sample size calculation indicated that 18 patients per group were required. Thus, 20 patients per group (*n* = 20) were planned for enrollment (α = 0.05, power = 0.80). The calculated minimum sample size was 18 participants per group. Therefore, our goal is to recruit 20 patients per group. The results of this study should be able to determine the initial safety, feasibility, and effectiveness of RIC in aSAH patients, providing good guidance and reference for further conducting large-scale randomized double-blind clinical studies (REPAIR).

### Statistical analysis

The main analysis adopts the intention-to-treat set, and all randomized subjects are included in the original allocation group; the per-protocol set analysis (PP) is used as a sensitivity analysis supplement. Categorical variables are presented as frequencies and percentages. Group differences will be assessed using Fisher’s exact test because of the small sample size. Continuous variables are subjected to Shapiro–Wilk normality test, and normal distribution data are analyzed using independent sample t-test (mean ± standard deviation), while non-normal data are analyzed using Mann–Whitney U test (median [IQR]). Missing data will be handled using multiple imputation with five imputations. In addition, worst-case and best-case scenario analyses will be conducted as sensitivity checks to assess the robustness of the results. For clinical events such as death, the intention-to-treat principle (ITT) are adopted, and cases of loss to follow-up are classified as no-event outcomes. Statistical analysis will be conducted using the Windows version 26.0 of SPSS statistical software (located in Ammonk, New York, produced by IBM Corporation).

## Discussion

Although prior trials have evaluated RIC in the acute course of aSAH, their designs and endpoints differ materially from ours. Raval et al. conducted a prospective randomized pilot study initiating lower-limb RIC within 72 h of symptom onset and delivering 4 × 5-min cycles every other day up to day 14 or ICU discharge; the intervention proved feasible and safe (no lower-extremity deep vein thrombosis or neurovascular injury) (20). However, the intervention included patients treated by both clipping and endovascular coiling, resulting in a heterogeneous population with respect to the risk of delayed cerebral ischemia. Albrecht et al. subsequently applied upper-limb RIC (3 × 5-min once daily for 10 days) after aneurysm securing, but RIC did not reduce vasospasm or infarctions (21). The dose of RIC they used was lower than that in previous studies (12–14), and this is likely the reason for the negative results. In contrast, our trial specifically enrolls patients within 24 h after microsurgical clipping, uses a standardized, device-controlled unilateral lower-limb protocol (5 × 5-min, twice daily for 7 days) to minimize operator variability, and adopts blinded functional outcomes.

In 2021, Zhu et al. showed that microsurgical clipping surgery for ruptured aneurysms had a higher adverse outcome and vasospasm risk compared to endovascular interventional treatment (9). Therefore, we plan to select aSAH patients within 24 h after microsurgical clipping as the research subjects, hoping to increase the detection efficiency of the effectiveness of RIC in aSAH patients. In addition, currently, large-scale randomized controlled clinical trials of RIC applied to ischemic stroke mostly select the upper limbs for treatment. However, our preliminary investigation suggested that upper-limb application was less practical in postoperative aSAH patients because of interference with intravenous lines and hemodynamic monitoring. Therefore, we select the lower limbs of aSAH patients for treatment to reduce the impact on the clinical treatment of patients.

This study has some limitations. Firstly, the RIC dose used in this study is determined based on the length of hospital stay of aSAH patients in clinical practice, although it is feasible in clinical practice, it has not been verified through dose exploration studies for the threshold of effect, and it may not be the optimal dose. Secondly, the subjects in this study are from two medical centers, and the population characteristics are relatively homogeneous, which may lead to selection bias and may affect the generalizability of the results. In addition, although randomization has balanced known confounding factors, there may still be baseline imbalance under a small sample size. Additionally, as the intervention involves limb ischemia, mild local discomfort or transient pain may occur. These aspects will be carefully monitored and reported to ensure patient safety and data integrity.
